# Utility of the signal intensity ratio of the spleen and myocardium (SMR) on T2-weighted short tau inversion recovery black-blood (T2-STIR BB) images compared to semi-quantitative analysis in patients with images with diffuse high T2 signal intensity, such as that with Takotsubo cardiomyopathy, cardiac amyloidosis, hypereosinophilic myocarditis, and apical hypertrophic cardiomyopathy

**DOI:** 10.1186/1532-429X-18-S1-Q40

**Published:** 2016-01-27

**Authors:** Ryosuke Aoki, Takatomo Nakajima, Yosuke Nakano, Reina Tonegawa, Keiko Masuoka, Makoto Muto

**Affiliations:** 1 Radiological Techniqucs, Saitama Cardiovasucular and Respiratory Center, Kumagaya, Japan; 2 grid.419430.bCardiology, Saitama Cardiovascular and Respiratory Center, 1696 Itai, Kumagaya-city, Japan

## Background

T2-weighted short tau inversion recovery black-blood (T2-STIR BB) images are widely used to evaluate edematous changes in the myocardium, such as that with acute myocardial infarction, myocarditis, and several cardiomyopathies, and visual assessment has been the main method to analyze T2-STIR BB images. Though T2 mapping may provide quantitative evaluation of T2 signal intensity, T2 mapping requires additional imaging and cannot accommodate analysis of conventional T2-STIR BB images.

## Methods

Among 376 consecutive patients who underwent cardiac magnetic resonance (MR) imaging examination using 1.5 Tesla MR system (Intera Achieva with 32-channel coil, Philps Medical Systems, Netherlands) between January 1, 2013 through December 31, 2014, we identified 22 cases with left ventricular ejection fraction exceeding 55% and no late gadolinium enhancement as our normal group to determine the normal range of the signal intensity ratio of the spleen and myocardium (SMR) on T2-STIR BB images. Furthermore, we evaluated the SMR of T2 -STIR BB images that showed diffuse high signal intensity, including Takotsubo cardiomyopathies (3 cases), cardiac amyloidosis (2 cases), hypereosinophilic myocarditis (one case), and apical hypertrophic cardiomyopathies (6 cases).

## Results

The SMR of the normal group was 0.477 ± 0.098, so we determined the normal range of the SMR less than 0.58 (as mean + 2 standard deviations [SD]). The figure shows SMR findings of the 12 patients who demonstrated diffuse high signal intensity on T2-STIR BB images. The results of SMR that showed diffuse high signal intensity were Takotsubo cardiomyopathies; 0.68, cardiac amyloidosis; 0.77, hypereosinophilic myocarditis; 0.97, and apical hypertrophic cardiomyopathies; 0.64, respectively. Diagnostic ability of diffuse high signal intensity on T2-STIR BB was better for SMR (100%; 12/12) than visual assessment (75%; 9/12).

## Conclusions

The SMR on conventional T2-STIR BB images may provide quantitative results in patients who show diffuse high signal intensity on T2-STIR BB images and offer better diagnostic ability than visual analysis of those images.

**Figure 1:**
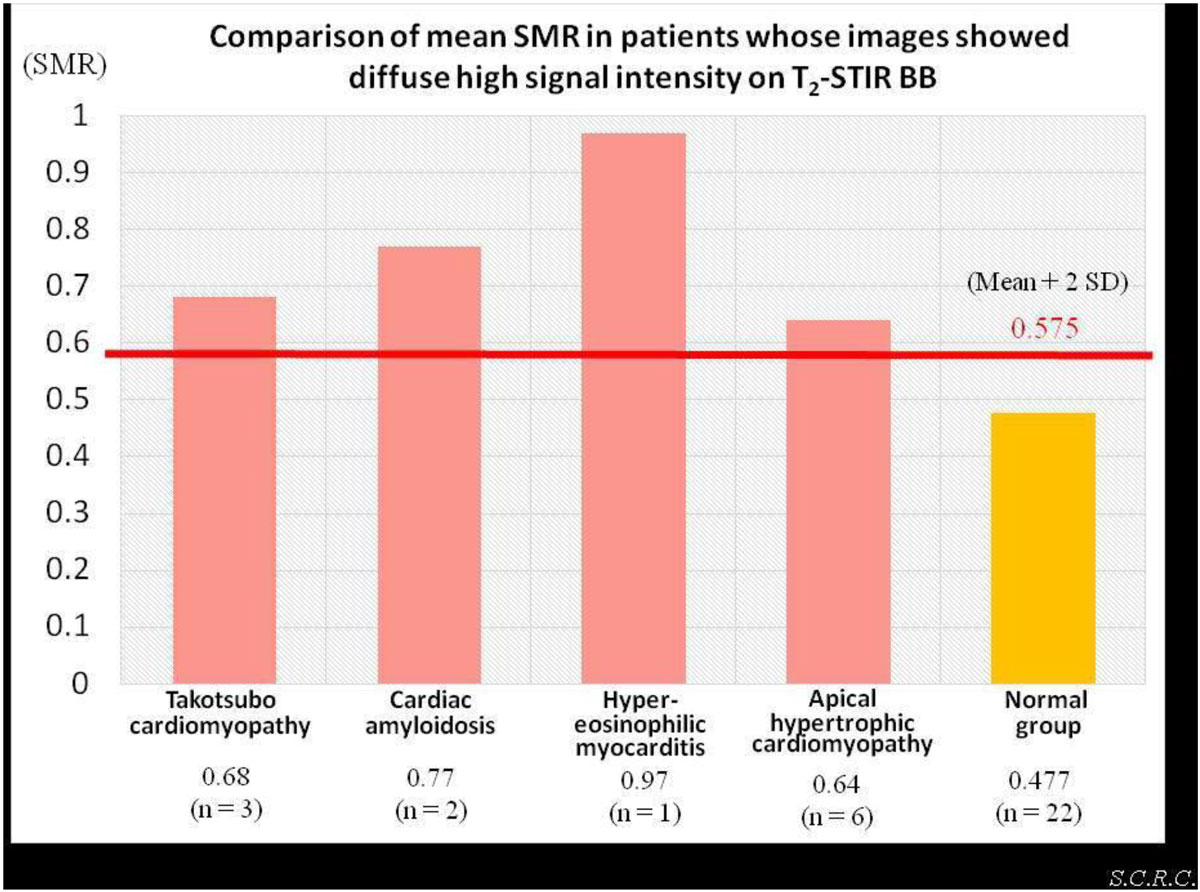
Figure 1

